# Eukaryotic initiation factor 3, subunit C silencing inhibits cell proliferation and promotes apoptosis in human ovarian cancer cells

**DOI:** 10.1042/BSR20191124

**Published:** 2019-08-05

**Authors:** Fang Wen, Zhang-Ying Wu, Lei Nie, Qi-Zhu Zhang, Yuan-Kun Qin, Zun-lun Zhou, Jin-Jian Wu, Xing Zhao, Jun Tan, Darrell Sawmiller, Dan Zi

**Affiliations:** 1 Department of Obstetrics and Gynecology, Affiliated Hospital of Guizhou Medical University, Guiyang 550004, Guizhou, China; 2 National and Guizhou Joint Engineering Laboratory for Cell Engineering and Biomedicine Technique, Center for Tissue Engineering and Stem Cell Research, Guizhou Province Key Laboratory of Regenerative Medicine, Guizhou Medical University, Guiyang 550004, Guizhou, China; 3 Department of Psychiatry and Behavioral Neurosciences, Morsani College of Medicine, University of South Florida, 3515 E Fletcher Ave. Tampa 33613, FL, U.S.A.

**Keywords:** apoptosis, eIF3c, IPA, ovarian cancer cells,, proliferation

## Abstract

Ovarian cancer remains the leading cause of death among all gynaecological cancers, illustrating the urgent need to understand the molecular mechanisms involved in this disease. Eukaryotic initiation factor 3c (EIF3c) plays an important role in protein translation and cancer cell growth and proliferation, but its role in human ovarian cancer is unclear. Our results showed that EIF3c silencing significantly up-regulated 217 and down-regulated 340 genes. Ingenuity Pathway Analysis (IPA) indicated that the top differentially expressed genes are involved in ‘Classical Pathways’, ‘Diseases and Functions’ and ‘Networks’, especially those involved in signalling and cellular growth and proliferation. In addition, eIF3c silencing inhibited cellular proliferation, enhanced apoptosis and regulated the expression of apoptosis-associated proteins. In conclusion, these results indicate that by dysregulating translational initiation, eIF3c plays an important role in the proliferation and survival of human ovarian cancer cells. These results should provide experimental directions for further in-depth studies on important human ovarian cancer cell pathways.

## Introduction

Ovarian cancer remains the leading cause of death in all gynaecological malignancies [1]. Currently, surgery is the main treatment for early ovarian cancer. Surgery is usually supplemented with chemotherapy to provide comprehensive treatment. Approximately 80% of patients with advanced ovarian cancer still experience tumour progression and recurrence, which eventually leads to death due to acquired resistance to platinum-based anti-tumour drugs [2]. This poor prognosis suggests an urgent need to identify new molecular mechanisms involved in ovarian cancer and to find novel molecular targets for the treatment of this disease. Within the last decade, the molecular underpinnings of many forms of cancer have been extensively investigated and revealed, which has facilitated the development of tailored therapies [3].

Eukaryotic initiation factors (eIFs), which regulate the initiation of translation, protein synthesis, cell growth and proliferation, are aberrantly expressed in several human tumours [4–9]. EIf3c, an important member of the general translation initiation complex, consisting of 913 amino acids and located at 16p11.2, is a housekeeping gene in the cytoplasm. eIF3c has been demonstrated to be essential for cell proliferation in numerous human tumours, including hepatocellular carcinoma, glioma and colon cancer [10–12]. However, little is known about the role of eIF3c in human ovarian cancer.

In recent years, the molecular pathogenesis of various tumours has been investigated at the molecular level using various bioinformatics approaches, including biochip and sequence analysis, biological data clustering and pathway analysis, providing novel research ideas for further study [13–15]. In the present study, to explore the molecular pathways potentially linked to ovarian cancer, changes in gene expression in eIF3c-downregulated SKOV3 human ovarian cancer cells were investigated using GeneChip microarray and pathway analysis. Canonical pathways linked to ovarian cancer were identified and ranked. The findings presented here provide new insights into the biological role of the *eIF3* gene in human ovarian cancer and provide novel ideas for ovarian cancer therapy targetting eIF3-related molecular pathways.

## Materials and methods

### SKOV3 and HO-8910 cell culture and eIF3c silencing

SKOV3 and HO-8910 cells (American Tissue Culture Collection, Manassas, VA, U.S.A.) were cultured in a humidified 37°C incubator with a 5% CO_2_ atmosphere in Dulbecco’s modified Eagle’s medium (DMEM, Gibco, U.S.A.) supplemented with 10% FBS, 100 U/ml penicillin, and 100 ng/ml streptomycin. EIF3c-siRNA (5′-GAC CAT CCG TAA TGC CAT GAA-3′) and scrambled siRNA (5′-TTC TCC GAA CGT GTC ACG T-3′) was inserted into the lentiviral shRNA expression vector pGCSIL-GFP using the Lentivector Expression Systems (GeneChem, Shanghai, China). The identities of the generated siRNA-expressing vectors were confirmed by DNA sequencing. Human renal epithelial 293T cells were infected with eIF3c-shRNA and scrambled siRNA plasmids (negative control) to generate eIF3c-shRNA and scrambled siRNA lentivector particles. Human ovarian cancer SKOV3 and HO-8910 cells were then infected with eIF3c-shRNA and scrambled shRNA lentiviral particles (negative control) at a multiplicity of infection (MOI) of 100. After 72 h of infection, the expression of GFP was observed by fluorescence microscopy to identify transfected cells. After 120 h of infection, the cells were harvested to determine the eIF3c knockdown efficiency by real-time quantitative PCR (RT-qPCR).

### RT-qPCR detection of eIF3c expression

The RT-qPCR analysis was performed utilizing an RT-qPCR kit (SYBR Green I) according to the manufacturer’s instructions (Thermo Fisher Scientific, Waltham, MA). cDNA (1 μg) was used as a template for PCR with the following primers: eIF3c forward, 5′-CCATCCTCTGCCACATCTACC-3′ and reverse, 5′-CCACCTTCTCCTGCTCCTG-3′, product size of 294 bp; cysteine-rich, angiogenic inducer, 61 (CYR61) forward, 5′-AGACCCTGTGAATATAACTCCA-3′ and reverse, 5′-AATTGCGATTAACTCATTGTTT-3′, product size of 300 bp; ANKRD1 forward, 5′-AGTAGAGGAACTGGTCACTGG-3′ and reverse, 5′-TGTTTCTCGCTTTTCCACTGTT-3′, product size of 201 bp; RAP1A forward, 5′-CAAGCTAGTAGTCCTTGGTTCAG-3′ and reverse, 5′-GGAATCTTCTATCGTTGGGTCAT-3′, product size of 106 bp; and GAPDH forward, 5′-TGACTTCAACAGCGACACCCA-3′ and reverse, 5′-CACCCTGTTGCTGTAGCCAAA-3′, product size of 121 bp. PCR products were separated on a 2% agarose gel, stained with Ethidium Bromide and analysed by UV imaging.

### Ingenuity Pathway Analysis

Total RNA from eIF3c shRNA- or scrambled siRNA-infected SKOV3 cells was analysed by a NanoDrop 2000 (Thermo) and an Agilent Bioanalyzer 2100 (Santa Cruz, CA) for quantity and quality. Reverse transcription, double-stranded DNA template conversion and transcription for RNA synthesis and labelling was performed using a GeneChip 3′IVT Express kit (Affymetrix Inc., Santa Barbara, CA) per the manufacturer’s instructions. Transcribed RNA was hybridized, washed and stained with a GeneChip Hybridization Wash and Stain kit (Affymetrix Inc., Santa Barbara, CA), and microarray analysis was performed using a Prime View Human Gene Expression Array (Thermo Fisher). Significantly, differentially expressed genes between SKOV3 cells treated with eIF3C-shRNAs and those treated with scrambled shRNA, defined as genes with an absolute log-transformed fold change (abs(logFC)) > 1.5 (*P*<0.05), were identified by array scanning using a GeneChip Scanner 3000. The results were analysed by Ingenuity Pathway Analysis (IPA) (Qiagen, Venlo, NL) using the ‘core analysis’ function to interpret the data from the perspective of biological processes, pathways, and networks. Canonical pathway (CP) analysis was used to identify function-specific genes significantly present within the identified networks. Z-scores were calculated to show the extent to which each pathway was activated or suppressed upon eIF3c silencing. A Z-score > 2 means the pathway was significantly activated, while a Z-score < −2 represents a significant inhibition of the pathway.

### Cell proliferation assay (MTT assay and Celigo)

#### MTT assay

SKOV3 cells expressing eIF3c- or scrambled shRNA were cultured for 72 h. Cell proliferation was then assessed with MTT staining (Genview, Houston, TX). In brief, the cells were seeded in 96-well plates at 2000 cells per well and cultured for 5 days. The MTT reagent was then added to the cells at 5 mg/ml, and after 4 h, the absorbance at 490/570 nm was measured using a microplate reader.

#### Celigo assay

SKOV3 cells were cultured in 96-well plates at a density of 2000 cells per well. From the second day after plating, the number of green fluorescent cells was determined every 24 h for a total of 120 h using a Celigo Image Cytometer (Nexcelom, Lawrence, MA) as a measure of cell proliferation.

#### Transwell assay

To transfect the cells, add 100–200 μl of the cell suspension to the Transwell chamber (Corning, U.S.A.), refer to the instructions. Five hundred microlitres of medium containing FBS or chemokine is usually added to the lower chamber of the 24-well plate. The amount of different plates is different. Please refer to the instruction manual for details. Conventional culture for 48 h (mainly depending on the invasive ability of cancer cells). The supernatant was removed, Crystal Violet stained and the cells in the upper chamber were removed and photographed by microscopy.

### Evaluation of apoptosis

Apoptosis was assessed using an eBioscience™ Annexin V-APC Apoptosis Detection kit (Thermo Fisher). Briefly, cells were transfected as described above, incubated for 5 days, resuspended in binding buffer at a density of 1 × 10^6^ cells/ml, transferred (100 μl) to FACS tubes and stained with Annexin V. Cells were mixed gently in a dark room for 15 min at room temperature and then analysed for apoptosis with an apoptosis detection kit (eBioscience 88-8007, U.S.A.).

### Western blotting

After eIF3c or scrambled shRNA transfection, the cells were collected and lysed with cell lysis buffer (Sigma). The antibody dilution ratio was as follows: eIF3c (1:500, Cell Signaling Technology, CST), Bax (1:1000, CST), Bcl2 (1:1500, Abcam), cleaved caspase 3 (1:1000, Cell Signaling Technology, CST) and β-actin (1:5000, CST).

### Statistical analysis

Data are presented as the mean ± SEM from at least three independent experiments. A paired *t* test was used to analyse differences in the levels of mRNA expression and cellular proliferation and apoptosis between eIF3c-silenced and control cells using GraphPad Prism 5. A *P*-value <0.05 was considered statistically significant.

## Results

### Microarray analysis of the SKOV3 ovarian cancer cell line after eIF3c gene silencing

**Figure 1:**
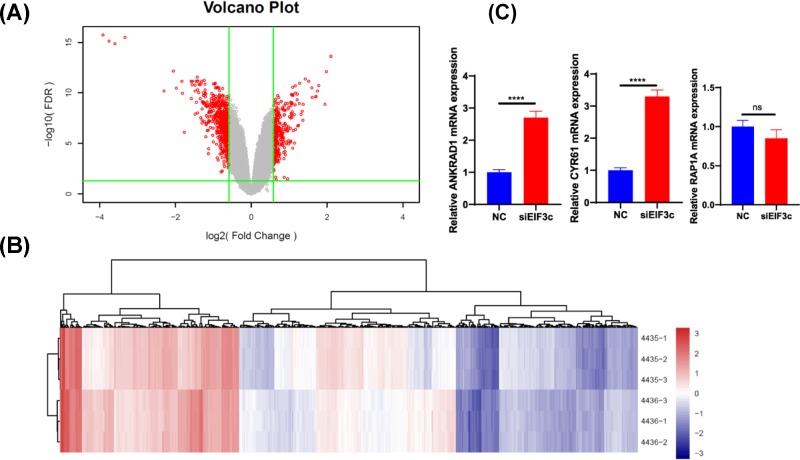
The clustering of differentially expressed genes in SKOV3 cells after elF3 silencing as visualized by volcano maps and heatmap analysis (**A**) All the differential genes shown by volcano maps; the red part means significant difference. (**B**) The significantly different genes shown by volcano maps. (**C**) RT-PCR analysis the ANKRD1, RAP1A and CYR61 expression. The data showed that the expression level of ANKRD1 and CYR61 was significantly higher in the eIF3c-silenced group than the negative control group (*P*<0.05). There was no significant difference in RAP1A expression between the silenced group and the negative group (*P*>0.05). ***P<0.001, ****P<0.0001.

After shRNA lentivirus infection, the expression of EIF3C in the experimental group was inhibited (Supplementary Figure S1). Affymetrix Human GeneChip prime view analysis revealed that the expression of 557 genes was significantly altered by eIF3c silencing (Z-score > 1.5, *P*<0.05) (Supplementary Table S1). Among these genes, 217 exhibited up-regulated expression and 340 genes exhibited down-regulated expression (Figure 1A,B). In Figure 1A, the horizontal coordinate is the difference multiple (base 2 logarithmic transformation), the vertical coordinate is the significant FDR (base 10 logarithmic transformation), the red dots are the significantly differentially expressed genes screened with | fold change | > 1.5 and FDR < 0.05 as the standard, and the grey dots are the other genes with no significant difference in expression. Figure 1B is a heatmap of the hierarchical clustering of the KD and NC samples using the expression profiles of differentially expressed genes selected with | fold change | > 1.5 and FDR < 0.05 as the selection criteria. Each row represents a sample, and each column represents a differentially expressed gene; the left tree structure is based on the expression profile of the differentially expressed genes, the aggregation or the classification of all samples; the upper tree structure represents the aggregation of the expression patterns of the differentially expressed genes; the red colour indicates that the expression level of the genes is relatively up-regulated, and the blue colour indicates that the expression level of the genes is relatively down-regulated. There was no significant change in the expression of white coloured genes. The top ten genes significantly up- and down-regulated are listed in Table 1. The candidate genes *THBS1, RAP1A, CYR61, ADAMTS1, TUFT1, CFL2* and *ANKRD* were screened and verified. The data showed that the expression level of ANKRD1 and CYR61 was significantly higher in the eIF3c-silenced group than the negative control group (*P*<0.05). There was no significant difference in RAP1A expression between the silenced group and the negative group (*P*>0.05) (Figure 1C).

**Table 1:** The top ten down-regulated and up-regulated proteins identified by Affymatrix Human GeneChip analysis

Entrez	Gene symbol	Gene title	*P*-value	Fold change
5906	RAP1A	RAP1A, member of RAS oncogene family	1.68E-20	−10.07126733
1073	CFL2	Cofilin 2 (muscle)	3.34E-14	−3.936654227
9993	DGCR2	DiGeorge syndrome critical region gene 2	1.42E-13	−3.513713755
8663	EIF3C	Eukaryotic translation initiation factor 3 subunit C	2.28119E-08	−3.392079541
728689	EIF3CL	Eukaryotic translation initiation factor 3 subunit C-like	2.28119E-08	−3.392079541
7851	MALL	mal, T-cell differentiation protein-like	2.52617E-15	−2.995707293
10163	WASF2	WAS protein family member 2	7.72152E-13	−2.987502665
221476	PI16	Peptidase inhibitor 16	7.58374E-14	−2.797516551
6443	SGCB	Sarcoglycan β	9.64507E-11	−2.787638207
9500	MAGED1	MAGE family member D1	2.31339E-12	−2.730496549
574040	SNORA6	Small nucleolar RNA, H/ACA box 6	2.75875E-09	2.375371779
147947	ZNF542P	Zinc finger protein 542, pseudogene	3.75414E-12	2.414514845
22943	DKK1	Dickkopf WNT signaling pathway inhibitor 1	6.23718E-15	2.424935992
7286	TUFT1	Tuftelin 1	1.00731E-10	2.490266253
9510	ADAMTS1	ADAM metallopeptidase with thrombospondin type 1 motif 1	3.62238E-11	2.53994396
7057	THBS1	Thrombospondin 1	9.40453E-16	2.681233132
11096	ADAMTS5	ADAM metallopeptidase with thrombospondin type 1 motif 5	4.60691E-13	3.251299968
1490	CTGF	connective tissue growth factor	7.83564E-14	3.357048391
27063	ANKRD1	Ankyrin repeat domain 1 (cardiac muscle)	5.07223E-12	3.846314326
3491	CYR61	Cysteine-rich, angiogenic inducer, 61	3.19733E-18	4.288143939

### Bioinformatics analysis of differentially expressed genes

The association of the differentially expressed genes induced by eIF3c silencing was further analysed using IPA software (Qiagen), which showed that these genes are involved in ‘*Classical Pathways*’, ‘*Disease and Function*’ and ‘*Networks*’.

#### Classical pathway analysis

**Figure 2:**
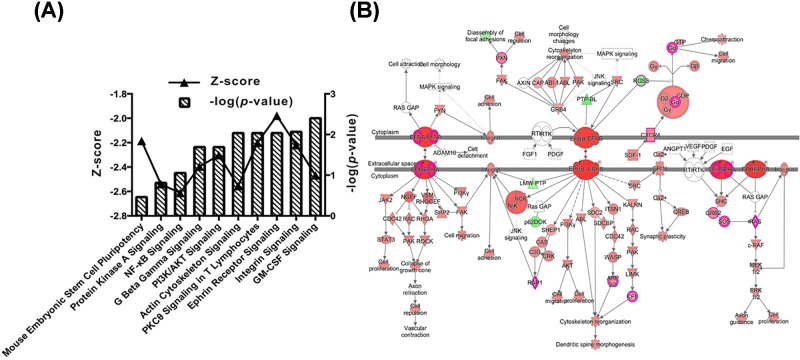
Differentially expressed ‘classical pathways’ after elF3 silencing as determined by iterative IPA (**A**) Top nine ‘classical pathways’ suppressed by elF3 silencing are shown. Left-vertical axis indicates log of the calculated *P*-value. Right-vertical axis indicates the Z-score value. (**B**) The Z-score is negative, which indicates the pathways decreased after elF3 silencing. When the Ephrin Receptor signaling was inhibited, other relative pathways were also affected.

A classical pathway analysis indicated that nine classical pathways, most notably Actin Cytoskeleton Signalling, PCK8 Signalling in T Lymphocytes, Ephrin Receptor Signalling and Integrin Signalling, were significantly suppressed after eIF3c silencing (Z-score = −2.121; Figure 2A). Furthermore, when Ephrin Receptor Signalling was inhibited, other relative pathways, particularly those involved in cell proliferation and movement, were also affected (Figure 2B).

#### Disease and function analysis

**Figure 3:**
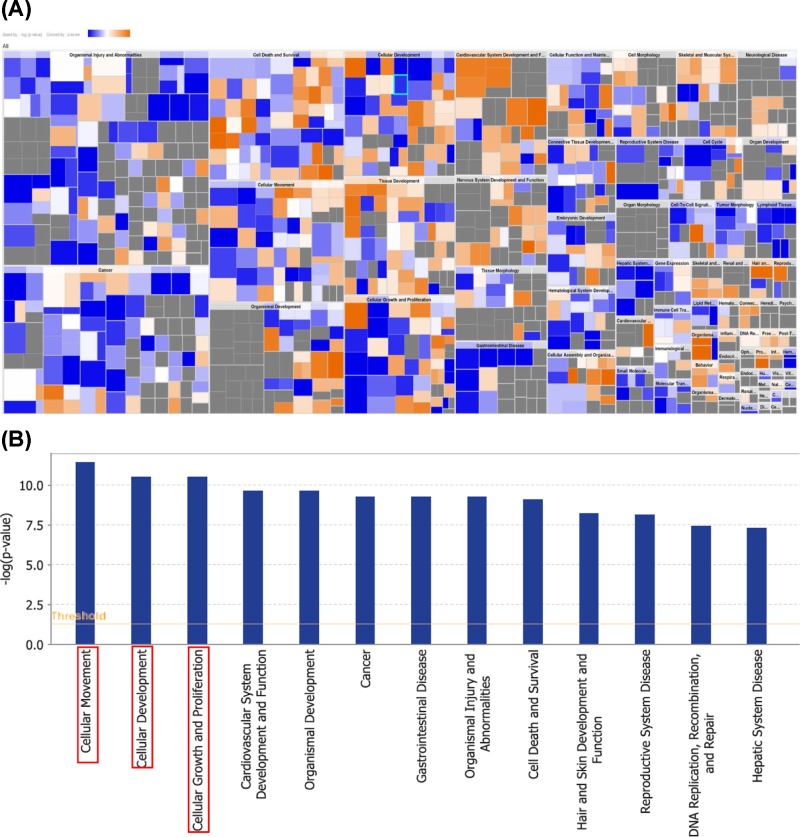
Diseases and functions analysis (**A**) Colour-coded heatmap analysis of differentially expressed genes after elF3 silencing, clustered according to functions and diseases. Orange indicates up-regulated gene expression; grey indicates down-regulated gene expression and blue indicates unidentified genes. (**B**) The most statistically significant functions altered by elF3 silencing are shown.

The top three disease and function differentially expressed gene clusters associated with eIF3c silencing were *Organismal Injury and Abnormalities, Cancer* and *Cell Death and Survival* (Figure 3A). Significantly activated diseases or functions included cell–cell contact and significantly suppressed diseases or functions: migration of microvascular endothelial cells (Supplementary Table S3). According to the function of the differentially expressed genes, the diseases were further classified into subcategories, particularly those associated with *Cellular Movement*, C*ellular Development* and *Cellular Growth and Proliferation* (Figure 3B).

#### Network analysis

To further understand these differentially expressed genes, they were subjected to network analysis by IPA. The database attributed general cellular functions to each of the networks, which were determined by interrogating the IPA knowledgebase for relationships between the genes in the network and the cellular functions they impact. The analysis of the genes identified above revealed six significant genetic networks (score ≥ 20.0), as listed in Supplementary Table S2. The top scoring networks among the diseases and functions included Cellular Movement, Cellular Development, Cellular Growth and Proliferation, Cardiovascular System Development and Function, Organismal Development, Cancer, Gastrointestinal Disease, Organismal Injury and Abnormalities, Cell Death and Survival, Hair and Skin Development and Function, Reproductive System Disease, DNA Replication, Recombination, and Repair, Hepatic System Disease, Cell Cycle, Embryonic Development, Organ Development, Tissue Development, Cellular Assembly and Organization, Cellular Function and Maintenance and Cell-To-Cell Signalling and Interaction.

### Knockdown of EIF3c suppresses cellular proliferation and increases apoptosis of SKOV3 and HO-8910 cells

**Figure 4:**
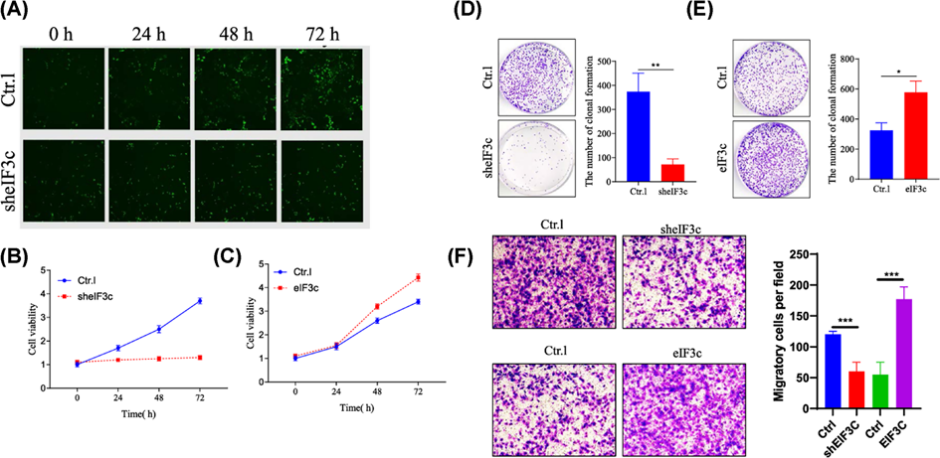
SKOV3 cell growth after elF3 silencing Cell growth was measured everyday for 5 days by GFP expression analysis, Cellgro cytometry and MTT staining. (**A**) Images of GFP expression in control and elF3 silenced SKOV3 cells over 5 days as observed by flourescence microscopy (100×). (**B**) The cell proliferation analyzed by MTT assay at knockdown elF3. (**C**) Overexpression of elf3, the cell proliferation analyzed by MTT assay. (**D**) The cell clonal formation analyzed at knockdown elF3. (**E**) Overexpression of elf3 in the cell clonal formation analysis. The proliferation of cells was significantly inhibited by eIF3C silencing. (**F**) The transwell assay showed that the invasive ability of cells decreased after silencing eIF3C. *P<0.05, **P<0.01, ***P<0.001, ****P<0.0001.

**Figure 5:**
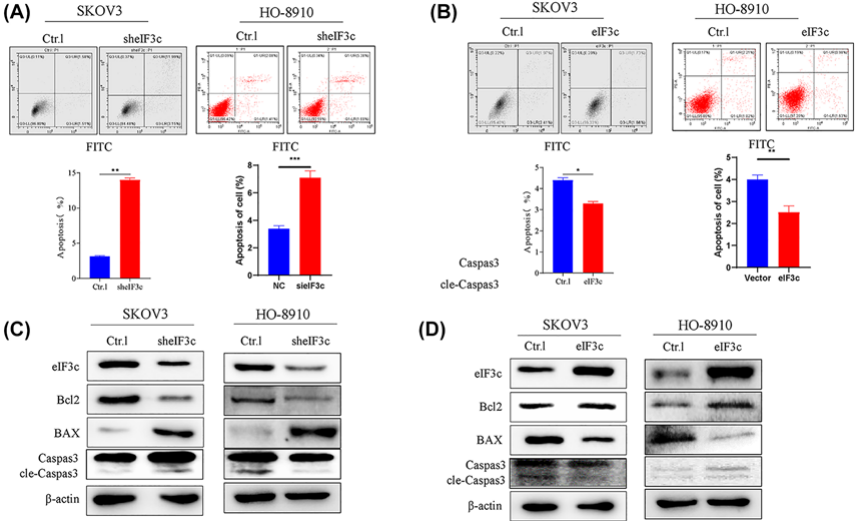
Silencing eif3c promoted SKOV3 and HO-8910 cells apoptosis (**A**) Silencing of elF3 increased SKOV3 and HO-8910 apoptosis as evaluated by flow cytometery. (**B**) Overexpression of elF3 suppressed SKOV3 and HO-8910 apoptosis as evaluated by flow cytometery. (**C**) The expression of apoptosis protein (Bcl2, Bax, Caspase 3, cle-Caspase 3) at silencing of elF3. (**D**) The expression of apoptosis protein (Bcl2, Bax, Caspase 3, cle-Caspase 3) at overexpression of elF3. Taken together, down-regulation of eif3c promoted apoptosis in SKOV3 and HO-8910 cells. *P<0.05, **P<0.01, ***P<0.001, ****P<0.0001.

The effect of eIF3c silencing on the proliferation rate was determined using MTT and Celigo assays. While the density of SKOV3 and HO-8910 cells increased over 5 days, eIF3c silencing reduced SKOV3 and HO-8910 density after 3 days of culture, as determined by GFP fluorescence microscopy (Figure 4A) as well as MTT analysis (Figure 4B,C) and a colony formation assay (Figure 4D,E). Our data showed that knockdown of eIF3c inhibited cell growth; however, cell growth was promoted when eIF3c was overexpressed. The transwell assay showed that the invasive ability of cells decreased after silencing eIF3C (Figure 4F). In addition, eIF3c silencing increased apoptosis (Figure 5A). However, increased eIF3c expression suppressed apoptosis (Figure 5B), as determined by flow cytometry of annexin V. Western blotting analysis of Bcl2, Bax and caspase3, cleaved-caspase3 activity (Figure 5C,D) showed that eIF3c inhibits the expression of apoptosis proteins. Therefore, the *eIF3c* gene mediates the proliferation and growth of SKOV3 and HO-8910 cells. Taken together, down-regulation of eif3c promoted apoptosis in SKOV3 and HO-8910 cells.

## Discussion

Ovarian cancer is the most lethal of malignant gynaecological tumours. Cancer cell proliferation and metastasis are critical for tumour progression and can lead to death of cancer patients. eIFs have been found to be aberrantly expressed in several human tumours [16]. As one of the most important initiation factors, eIF3 plays vital roles in translation initiation [17], as well as tumorigenesis and cell proliferation [18,19]. However, there is currently little to no research regarding the role of eIF3c in ovarian cancer. Therefore, we analysed differential gene expression by GeneChip microarray, the functions and networks of differentially expressed genes by IPA, and cell proliferation and apoptosis after silencing of eIF3c in the SKVO3 ovarian cancer cell line. The present study investigated the expression and function of eIF3c in ovarian cancer.

The Affymetrix GeneChip expression microarray is a gene chip that analyses human genome information. This GeneChip is more accurate, sensitive and reliable than RT-PCR and is one of the most widely applied gene expression microarrays. IPA is online integrated analysis software that can contribute to the understanding of molecular interaction networks by establishing an experimental visualization system. In IPA, the input data are the differential gene expression results of the chip analysis.

Hierarchical clustering analysis involves the grouping and classification of differentially expressed genes by sample and gene identity based on the similarity of gene expression data obtained from the biochip. Based on this analysis, we detected that gene expression profiles were similar within experimental and control groups but different between groups, reflecting the effects of eIF3c silencing on the expression of specific genes. In addition, we detected genes with similar functions or within the same biological pathway based on similar expression patterns. In the present study, RAP1A (a member of the RAS oncogene family) was the most clearly down-regulated gene, while CYR61 was the most clearly up-regulated gene after eIF3c silencing. RAP1A is a molecular switch that activates downstream MAPK signalling pathways and promotes the expression of several target molecules that participate in the development of tumours and other diseases [20]. The effects of RAP1 signalling-related molecules on cell proliferation and invasion have been repeatedly observed, and RAP1 also participates in the regulation of telomere structure [21]. Somatic cells do not display detectable telomerase activity, but telomerase activity is tightly regulated and seen mainly in germ cells, stem cells and some immune cell types that are highly proliferative. Telomerase reverse transcriptase promotes cancer cell proliferation by augmenting tRNA expression [22]. Evidence has suggested that telomerase reverse transcriptase can modulate the expression of various genes, including the targets of Wnt/β-catenin and NFκB signalling, which affect cancer progression and tumorigenesis [23,24]. CYR61 mediates many biological functions, including cell migration, proliferation, apoptosis and differentiation [25].

Pathway analysis by the iterative IPA software program identified several differentially expressed ‘classical’ signalling pathways. Notably, the ephrin receptor signalling pathway was significantly inhibited. It has been determined that the Eph/ephrin system plays an important role in embryonic development and has a slight effect on the physiology of adults [26]. In addition, Eph/ephrin is overexpressed in cancer stem cell regeneration and metastasis development [27]. In this pathway, EPHA and EPHB, which promote the migration and invasion of cancer cells, are particularly up-regulated [28]. In addition, based on our gene expression analysis, we identified the diseases and functions of these differentially expressed genes. The significantly up-regulated genes function in *Cell–Cell Contact*, and the significantly down-regulated genes function is *Migration of Microvascular Endothelial Cells*. Cell–cell contact can induce the differentiation of stem cells [29]. The migration of microvascular endothelial cells is closely related to the proliferation of tumour cells [30]. The differentially expressed genes have significant effects on Embryonic Development, Organ Development, Organ Morphology, Cell Morphology, Cellular Assembly, Cellular Function and so on.

In the present study, we detected differences in gene expression, biological processes, proliferation and apoptosis in the human ovarian cancer cell line SKOV3 after silencing of eIF3c. It is reasonable to deduce that eIF3c, which regulates translation initiation, plays an important role in the proliferation and survival of human ovarian cancer cells. The present study provides an experimental direction for further in-depth analysis of the molecular mechanisms of ovarian cancer development.

## Supporting information

**Supplementary Figure S1:** 

**Supplementary Table S1:** 557 significant differentially differentially expressed genes after eIF3c silencing are shown in the following diagram

**Supplementary Table S2:** High-scoring networks (score > 20) identified by Ingenuity Pathway Analysis® in SKOV3 cells. The top six of 25 networks are represented here

**Supplementary Table S3:** The change of differential gene expression level can inhibit the activation of disease and function.

## Abbreviations

ANKRD1: Ankyrin Repeat Domain 1
CYR6: cysteine-rich, angiogenic inducer, 61
eIF: eukaryotic initiation factor
EPHA: EPH Receptor A
EPHB: EPH Receptor B
FDR: False discovery rate
GAPDH: Glyceraldehyde-3-Phosphate Dehydrogenase
IPA: Ingenuity Pathway Analysis
KD: Knock Down
NC: Negative Control
RAP1A: Member Of RAS Oncogene Family
RT-qPCR: real-time quantitative PCR
