# Supplementary material for: Eukaryotic initiation factor 3, subunit C silencing inhibits cell proliferation and promotes apoptosis in human ovarian cancer cells
Source: Biosci Rep. 2019 Aug 5;39(8):BSR20191124. doi: 10.1042/BSR20191124 (PMC6685053; doi:10.1042/BSR20191124)
Supplement: Supplementary file 1 [file bsr20191124_Supp1.pdf]

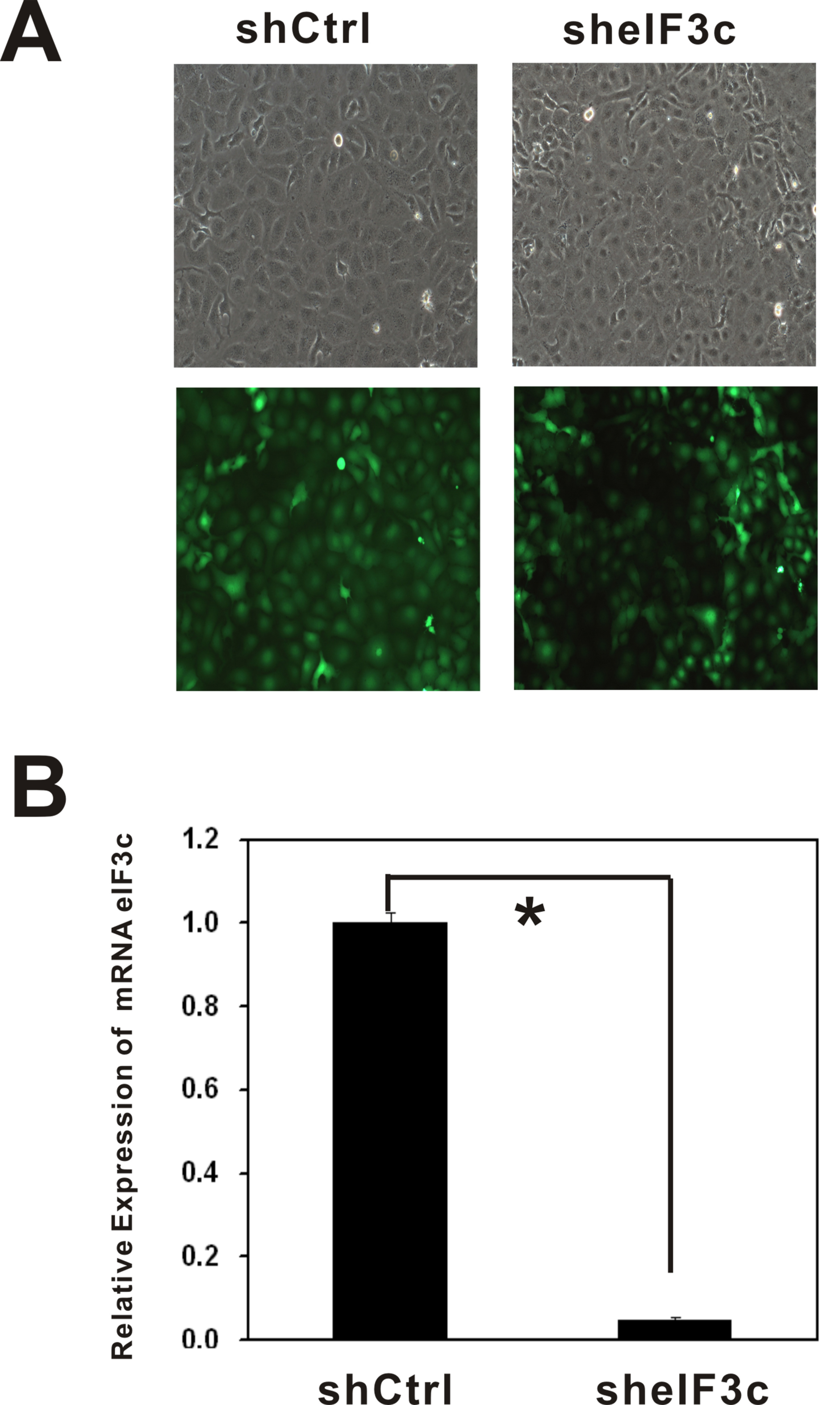

A
shCtrl
sheIF3c
B
Relative Expression of mRNA eIF3c
1.2
1.0
0.8
0.6
0.4
0.2
0.0
*
shCtrl
sheIF3c

**Table S1 557 significant differentially differentially expressed genes after eIF3c silencing are shown in the following diagram**

| | Gene Symbol | Absolute FC | *P*-value |
|---|---|---|---|
| 1. | ROPN1L | 1.518864671 | 8.03672E-06 |
| 2. | IGDCC4 | 1.538900548 | 1.99773E-08 |
| 3. | LETM2 | 1.548595119 | 8.60298E-08 |
| 4. | AHNAK | 1.692258543 | 2.62359E-13 |
| 5. | NAMPT | 1.705987016 | 5.44839E-11 |
| 6. | LARS2 | 1.621875787 | 3.81374E-08 |
| 7. | SURF4 | 1.502543404 | 5.10822E-11 |
| 8. | RAB31 | 2.065111961 | 1.83843E-10 |
| 9. | LEMD1 | 1.574634144 | 3.83051E-07 |
| 10. | KIRREL | 1.511013622 | 0.001063354 |
| 11. | KRT5 | 2.390904767 | 2.37061E-12 |
| 12. | PREPL | 1.849681237 | 1.4164E-09 |
| 13. | HSPA4L | 1.560364773 | 1.2704E-06 |
| 14. | LOC102724250 | 1.741692578 | 7.96928E-08 |
| 15. | SYDE1 | 1.634028712 | 8.70285E-09 |
| 16. | SHISA3 | 1.655633486 | 3.84144E-06 |
| 17. | FAM213B | 1.874284554 | 5.61632E-11 |
| 18. | GLUD2 | 1.626529137 | 2.79566E-12 |
| 19. | ATL3 | 1.578666761 | 1.48932E-12 |
| 20. | CTGF | 3.357048391 | 7.83564E-14 |
| 21. | C12orf76 | 1.57439768 | 2.13685E-08 |
| 22. | FAM83A | 1.577857225 | 3.77058E-08 |
| 23. | KDELC2 | 1.837681399 | 1.37389E-09 |
| 24. | RHOBTB1 | 1.580473304 | 4.37119E-06 |
| 25. | GMFB | 1.691855866 | 5.5481E-11 |
| 26. | SNORA2A | 1.602802502 | 6.21515E-05 |
| 27. | ZSCAN16 | 1.514057504 | 3.88013E-06 |
| 28. | MBOAT1 | 1.502894078 | 1.86202E-08 |
| 29. | TMEM80 | 1.648411766 | 7.83744E-05 |
| 30. | ALDH3A1 | 2.19937458 | 1.25582E-08 |
| 31. | SGK2 | 1.554348493 | 2.15395E-05 |
| 32. | FAM20B | 1.739681658 | 6.53716E-10 |
| 33. | NOP16 | 1.519247235 | 1.46651E-08 |
| 34. | PDSS1 | 1.515744583 | 2.80501E-06 |
| 35. | USB1 | 1.551047999 | 4.19777E-09 |
| 36. | PPP1R2 | 1.718762255 | 1.84117E-10 |
| 37. | BACE1 | 1.952713102 | 1.34254E-10 |
| 38. | AZIN1 | 1.972283123 | 6.70807E-12 |
| 39. | EPB41L5 | 1.508631025 | 1.46619E-06 |

| | | | |
|---|---|---|---|
| 40. | PIR | 1.529128873 | 4.82663E-10 |
| 41. | GCHFR | 2.407039805 | 3.26701E-12 |
| 42. | PPIF | 1.5465963 | 5.09238E-08 |
| 43. | WWC3 | 1.972725196 | 2.11673E-10 |
| 44. | SLC33A1 | 1.610386147 | 1.75497E-07 |
| 45. | DKK1 | 2.424935992 | 6.23718E-15 |
| 46. | TRIM2 | 1.612299765 | 3.11878E-08 |
| 47. | IFITM1 | 1.574506813 | 9.16998E-11 |
| 48. | HIST1H2AG | 1.723546235 | 1.51276E-07 |
| 49. | ATP6V0E1 | 1.513749692 | 3.59418E-10 |
| 50. | NADK2 | 1.755047406 | 1.6347E-10 |
| 51. | TGOLN2 | 2.599906967 | 9.35881E-15 |
| 52. | CBLL1 | 1.537120215 | 1.29674E-07 |
| 53. | FAM171B | 1.611748529 | 1.57487E-08 |
| 54. | ZBED2 | 1.577448968 | 3.72302E-10 |
| 55. | SLC44A2 | 1.540152634 | 3.80643E-07 |
| 56. | DTWD1 | 1.51684815 | 1.25881E-05 |
| 57. | HCN2 | 1.644231381 | 1.67438E-06 |
| 58. | DOCK2 | 1.806553525 | 3.04127E-07 |
| 59. | SEMA3F | 1.50052775 | 8.16369E-07 |
| 60. | BDH1 | 1.774197562 | 1.22917E-06 |
| 61. | TBC1D8 | 1.529588237 | 5.26249E-10 |
| 62. | RASSF2 | 1.503421979 | 2.965E-06 |
| 63. | ARL6IP5 | 1.515657033 | 5.90144E-10 |
| 64. | CSRP1 | 1.505170236 | 2.36213E-08 |
| 65. | MKNK2 | 2.360359594 | 1.47044E-15 |
| 66. | MRTO4 | 1.561385381 | 3.46643E-06 |
| 67. | MAGED1 | 2.730496549 | 2.31339E-12 |
| 68. | NBPF19 | 1.741692578 | 7.96928E-08 |
| 69. | MYPN | 2.133570822 | 2.0991E-06 |
| 70. | HPS3 | 2.053867814 | 6.59246E-12 |
| 71. | STBD1 | 1.508955227 | 1.54478E-06 |
| 72. | PXN | 1.52226198 | 2.80436E-06 |
| 73. | FECH | 2.459383415 | 1.38616E-09 |
| 74. | XDH | 1.55735013 | 1.90182E-07 |
| 75. | SORBS2 | 1.703883464 | 1.69387E-07 |
| 76. | GNAQ | 1.736749883 | 1.99344E-09 |
| 77. | MTMR9 | 1.646873794 | 2.05905E-08 |
| 78. | SPG20 | 1.792090299 | 1.43745E-09 |
| 79. | MAP3K7CL | 2.055320433 | 2.63319E-07 |
| 80. | PEX5 | 1.536814817 | 3.53743E-09 |
| 81. | PNO1 | 1.826539981 | 7.7386E-12 |
| 82. | ZFP36L2 | 1.534239099 | 7.60699E-11 |
| 83. | ARSJ | 1.786133605 | 2.82705E-09 |

| | | | |
|---|---|---|---|
| 84. | POLR3C | 1.571755342 | 8.89675E-07 |
| 85. | IPO11-LRRC70 | 1.769264815 | 1.24738E-10 |
| 86. | MPZL3 | 1.505076342 | 1.61328E-07 |
| 87. | CBSL | 1.561908565 | 1.40561E-09 |
| 88. | UBASH3B | 1.520486844 | 3.49283E-08 |
| 89. | ELF3 | 1.547286119 | 2.46349E-10 |
| 90. | RGS3 | 1.633319089 | 8.58552E-06 |
| 91. | ASAH2 | 1.533512578 | 4.15317E-05 |
| 92. | CPA4 | 1.707927419 | 7.1828E-09 |
| 93. | MYBL1 | 1.871917258 | 3.62679E-12 |
| 94. | IER3IP1 | 1.730841159 | 1.73545E-12 |
| 95. | SGCB | 2.787638207 | 9.64507E-11 |
| 96. | PCK2 | 1.562684645 | 1.04192E-07 |
| 97. | MZT1 | 2.115130483 | 2.38309E-13 |
| 98. | NABP1 | 2.162754634 | 1.32944E-08 |
| 99. | NOV | 1.805794012 | 2.41449E-10 |
| 100. | HN1L | 1.765605873 | 2.27705E-09 |
| 101. | PNRC1 | 1.552492896 | 1.8181E-09 |
| 102. | RFX2 | 1.529245467 | 8.91457E-06 |
| 103. | GGA2 | 1.52591366 | 6.05086E-05 |
| 104. | SNAPC1 | 1.5994989 | 3.1143E-12 |
| 105. | PIKFYVE | 1.565271951 | 3.14173E-09 |
| 106. | IFIT2 | 1.767581414 | 2.00852E-09 |
| 107. | TMEM185A | 2.139153485 | 5.15104E-11 |
| 108. | FZD1 | 1.997769311 | 3.82343E-12 |
| 109. | KLF13 | 1.633228521 | 4.11697E-09 |
| 110. | TNS2 | 1.525095938 | 3.31229E-07 |
| 111. | PI16 | 2.797516551 | 7.58374E-14 |
| 112. | SMCR8 | 1.525212225 | 5.22612E-08 |
| 113. | ADAMTS5 | 3.251299968 | 4.60691E-13 |
| 114. | WASF2 | 2.987502665 | 7.72152E-13 |
| 115. | ID2 | 2.103944527 | 1.29357E-11 |
| 116. | ZNF849P | 1.522803721 | 3.03158E-05 |
| 117. | SNCG | 1.584019395 | 1.51723E-05 |
| 118. | MIPOL1 | 1.607163458 | 8.46013E-06 |
| 119. | PXYLP1 | 2.063695335 | 3.04577E-12 |
| 120. | PHLDB2 | 1.575467507 | 5.13579E-05 |
| 121. | ABHD4 | 1.594694423 | 3.96204E-08 |
| 122. | RBM15B | 1.535505131 | 1.46436E-06 |
| 123. | GLUD1 | 1.664359014 | 3.37373E-12 |
| 124. | MMS22L | 1.551825853 | 1.12186E-08 |
| 125. | DNAJC6 | 1.753750279 | 8.39262E-08 |
| 126. | DDAH1 | 1.593596812 | 3.19074E-09 |
| 127. | LRRC6 | 1.544125486 | 1.33162E-06 |

| | | | |
|---|---|---|---|
| 128. | TICAM2 | 1.68130718 | 2.01978E-08 |
| 129. | KCTD2 | 1.502015812 | 1.24435E-05 |
| 130. | GPR180 | 1.701692073 | 1.65608E-10 |
| 131. | SFSWAP | 1.50396049 | 3.52408E-05 |
| 132. | NRP2 | 1.606877556 | 8.22436E-10 |
| 133. | JUN | 1.549790637 | 3.45042E-09 |
| 134. | CA12 | 1.985304086 | 1.00044E-11 |
| 135. | SPRYD7 | 1.638372419 | 2.36593E-10 |
| 136. | INPP4B | 1.642522728 | 3.91062E-06 |
| 137. | ATP8B2 | 1.741451145 | 1.84131E-06 |
| 138. | MMRN2 | 1.782015641 | 2.22027E-08 |
| 139. | ZNF680 | 1.657639171 | 3.62778E-07 |
| 140. | ZFP30 | 1.511362781 | 7.23479E-07 |
| 141. | RABL2A | 1.500742717 | 1.07781E-08 |
| 142. | FAM216A | 1.62852214 | 5.41315E-10 |
| 143. | FAM214B | 1.765846575 | 1.62241E-09 |
| 144. | ZNF880 | 1.514061002 | 3.3032E-06 |
| 145. | BDNF | 2.106186708 | 2.5669E-10 |
| 146. | CHGA | 2.228665347 | 1.33211E-08 |
| 147. | PTGDS | 1.888852439 | 3.70889E-10 |
| 148. | ZFP82 | 1.781151212 | 1.28984E-11 |
| 149. | LOC643923 | 1.579071685 | 1.11562E-05 |
| 150. | FAM47E-STBD1 | 1.508955227 | 1.54478E-06 |
| 151. | SAT1 | 1.573790313 | 8.76912E-10 |
| 152. | FAM25G | 1.750163858 | 2.18372E-12 |
| 153. | FOXS1 | 1.883096152 | 1.39967E-08 |
| 154. | FLOT1 | 1.556238669 | 1.29866E-08 |
| 155. | BSN | 1.530687736 | 4.31528E-05 |
| 156. | EEF1E1-BLOC1S5 | 1.535395154 | 1.87845E-09 |
| 157. | GNPNAT1 | 1.552808586 | 8.85249E-07 |
| 158. | POLD4 | 1.542406817 | 5.99545E-10 |
| 159. | NXNL2 | 1.52499023 | 6.06519E-09 |
| 160. | RCC1 | 1.56445844 | 1.04963E-07 |
| 161. | GUF1 | 1.506882229 | 9.34371E-06 |
| 162. | RAB40B | 1.685772671 | 6.71924E-11 |
| 163. | ANKRD1 | 3.846314326 | 5.07223E-12 |
| 164. | TIMP3 | 1.67739989 | 1.52226E-08 |
| 165. | CYR61 | 4.288143939 | 3.19733E-18 |
| 166. | CLIC3 | 2.321536559 | 5.10381E-14 |
| 167. | FNDC3B | 1.580655898 | 1.43889E-11 |
| 168. | ARPC5 | 2.5029567 | 5.12589E-15 |
| 169. | RPAP3 | 1.633262483 | 6.80374E-09 |
| 170. | WDR55 | 1.534405716 | 3.29897E-08 |
| 171. | PTPRF | 1.502234462 | 1.76491E-05 |

| | | | |
|---|---|---|---|
| 172. | TRIM31 | 2.351383736 | 9.10027E-10 |
| 173. | GADD45A | 1.547075209 | 1.43779E-12 |
| 174. | BASP1 | 1.663905309 | 7.80312E-11 |
| 175. | TM4SF4 | 1.693021155 | 1.00555E-05 |
| 176. | C6orf48 | 1.624125743 | 3.19792E-13 |
| 177. | BLOC1S5 | 1.535395154 | 1.87845E-09 |
| 178. | PKDCC | 1.521741527 | 2.85648E-06 |
| 179. | DMRT3 | 1.750899973 | 1.82132E-09 |
| 180. | BTF3L4 | 1.64182459 | 8.12284E-11 |
| 181. | HES2 | 1.79556349 | 8.40547E-09 |
| 182. | LEPROTL1 | 1.79006255 | 6.95133E-11 |
| 183. | SEC23A | 1.710616863 | 7.79619E-12 |
| 184. | PIM1 | 1.51387211 | 1.22125E-06 |
| 185. | CTNNB1 | 1.613824093 | 5.04152E-11 |
| 186. | SEC24D | 1.686477807 | 3.83315E-09 |
| 187. | F11R | 1.648187072 | 1.30719E-09 |
| 188. | ZNF542P | 2.414514845 | 3.75414E-12 |
| 189. | BICC1 | 1.754378455 | 2.81202E-08 |
| 190. | GPRIN3 | 1.522673545 | 0.000503932 |
| 191. | MYO5B | 1.574306742 | 1.58899E-06 |
| 192. | NQO1 | 1.631203376 | 1.0547E-11 |
| 193. | LTV1 | 1.554761549 | 5.91267E-09 |
| 194. | LAPTM5 | 1.790877512 | 7.00848E-08 |
| 195. | DBP | 1.768533238 | 3.37172E-10 |
| 196. | TMEM38B | 1.593942957 | 1.80493E-10 |
| 197. | ZBED6CL | 1.605853184 | 2.19318E-09 |
| 198. | CDC25A | 1.833355634 | 1.81154E-06 |
| 199. | ADK | 2.200172541 | 1.22564E-09 |
| 200. | ELMOD1 | 1.579071685 | 1.11562E-05 |
| 201. | THRB | 1.593063013 | 9.8989E-09 |
| 202. | MOB3B | 1.816792094 | 3.99984E-05 |
| 203. | TSR1 | 1.563403313 | 4.47453E-09 |
| 204. | PLK2 | 1.603328451 | 1.0336E-10 |
| 205. | ODC1 | 2.237731074 | 1.62303E-14 |
| 206. | CRYAB | 1.577299543 | 4.61494E-07 |
| 207. | ZNF492 | 1.522803721 | 3.03158E-05 |
| 208. | WNT10A | 1.592529393 | 3.27437E-09 |
| 209. | ITPRIPL2 | 1.848993304 | 6.89207E-11 |
| 210. | RRM2 | 1.609939715 | 2.66006E-09 |
| 211. | SOS2 | 1.613809178 | 1.11041E-08 |
| 212. | EIF4A2 | 1.635437546 | 6.53341E-09 |
| 213. | DHRS3 | 1.768802946 | 7.67788E-08 |
| 214. | RTN4 | 1.930195083 | 5.96819E-15 |
| 215. | KIF3B | 2.082442509 | 3.78084E-11 |

| | | | |
|---|---|---|---|
| 216. | DEPTOR | 2.180642595 | 2.35785E-06 |
| 217. | PLCB4 | 1.827992308 | 1.82677E-08 |
| 218. | PRUNE2 | 1.561443103 | 1.47557E-06 |
| 219. | CRIM1 | 1.537802255 | 4.47021E-05 |
| 220. | ESM1 | 1.767250643 | 3.65748E-08 |
| 221. | STX11 | 1.639932764 | 6.30938E-06 |
| 222. | MED14 | 2.15475922 | 8.83433E-12 |
| 223. | RBMS3 | 1.63031793 | 6.98836E-05 |
| 224. | HLA-DMB | 2.454558129 | 5.55029E-10 |
| 225. | ARRDC4 | 1.858359992 | 3.00492E-11 |
| 226. | SPARC | 1.569139192 | 4.9611E-07 |
| 227. | DPH3 | 1.859657149 | 1.06855E-08 |
| 228. | NCOA6 | 1.639781209 | 9.49804E-10 |
| 229. | HDHD2 | 1.844981905 | 8.03051E-10 |
| 230. | TIPIN | 1.566281293 | 3.46871E-08 |
| 231. | ZNF699 | 1.73476471 | 3.26038E-08 |
| 232. | ZSCAN12 | 1.643802152 | 3.46591E-06 |
| 233. | STK17A | 1.533728727 | 1.35792E-05 |
| 234. | SERPINA3 | 1.65288536 | 3.58817E-06 |
| 235. | MALL | 2.995707293 | 2.52617E-15 |
| 236. | MDM2 | 1.713457015 | 1.06601E-08 |
| 237. | CCBE1 | 1.527388077 | 1.17931E-08 |
| 238. | SNORA6 | 2.375371779 | 2.75875E-09 |
| 239. | CPEB3 | 1.510657563 | 1.88333E-06 |
| 240. | IDO1 | 1.555918687 | 5.81349E-12 |
| 241. | RBM24 | 1.860731639 | 1.78038E-09 |
| 242. | IL1RAP | 1.696333697 | 1.56501E-09 |
| 243. | PDZK1 | 1.815394804 | 1.04051E-07 |
| 244. | PCLO | 1.762382002 | 4.97099E-08 |
| 245. | ZMYM6NB | 2.540037858 | 8.06039E-13 |
| 246. | ANK1 | 1.747545477 | 7.73218E-07 |
| 247. | SLC1A4 | 1.794128637 | 2.44921E-08 |
| 248. | DMKN | 1.653737211 | 3.04774E-06 |
| 249. | ID4 | 1.68091876 | 9.53839E-13 |
| 250. | SYTL3 | 1.662410494 | 4.47974E-09 |
| 251. | NDUFAF6 | 1.506241744 | 7.93968E-08 |
| 252. | GPAT3 | 1.664855156 | 2.60286E-09 |
| 253. | MMP7 | 2.172124304 | 6.20085E-11 |
| 254. | MAPK9 | 2.340564734 | 3.32218E-09 |
| 255. | C14orf105 | 1.928768507 | 1.26024E-08 |
| 256. | SGK1 | 1.5034324 | 8.31402E-06 |
| 257. | PITPNA | 1.560682064 | 3.11701E-10 |
| 258. | ST6GALNAC4 | 1.876212625 | 2.15107E-08 |
| 259. | HES1 | 1.681684033 | 3.95693E-10 |

| | | | |
|---|---|---|---|
| 260. | VPS36 | 1.644037644 | 4.41802E-06 |
| 261. | RPUSD4 | 1.657880476 | 1.52682E-08 |
| 262. | ARL6 | 1.579593497 | 2.73155E-05 |
| 263. | BAMBI | 1.679521193 | 1.2793E-10 |
| 264. | SNORA44 | 1.812553192 | 7.24646E-06 |
| 265. | RAB29 | 1.552769121 | 2.70632E-09 |
| 266. | FOXO1 | 1.867679191 | 3.06899E-12 |
| 267. | WARS | 1.557285363 | 7.493E-11 |
| 268. | APPL2 | 1.620991658 | 1.28685E-08 |
| 269. | ADI1 | 1.500572821 | 7.47648E-10 |
| 270. | TSEN2 | 1.663720786 | 5.8905E-08 |
| 271. | SH3BP2 | 1.532039329 | 2.28067E-07 |
| 272. | PSKH1 | 1.590029281 | 1.89276E-08 |
| 273. | MAP3K3 | 1.557911557 | 3.95308E-09 |
| 274. | IGSF3 | 1.613082247 | 1.9891E-07 |
| 275. | USP5 | 1.5403626 | 3.95521E-08 |
| 276. | ANKRD46 | 1.591738491 | 2.20288E-07 |
| 277. | KIAA1217 | 1.55777478 | 8.77353E-07 |
| 278. | EIF3C | 3.392079541 | 2.28119E-08 |
| 279. | H6PD | 1.51568855 | 0.00033573 |
| 280. | ZNF429 | 1.583338807 | 0.013351 |
| 281. | NRBF2 | 1.6052411 | 2.74788E-08 |
| 282. | BOP1 | 1.555922282 | 2.72507E-11 |
| 283. | SLFN5 | 1.877873654 | 2.79142E-08 |
| 284. | SNX18 | 1.779563401 | 1.40692E-07 |
| 285. | GGCT | 1.531766792 | 2.39556E-08 |
| 286. | WHSC1 | 1.547593599 | 2.858E-08 |
| 287. | KLHL24 | 1.569160945 | 1.77909E-09 |
| 288. | EGR1 | 1.611379902 | 4.35639E-09 |
| 289. | EPHA4 | 1.7256621 | 7.92919E-07 |
| 290. | KIAA0895 | 1.774529633 | 1.54285E-07 |
| 291. | RALGDS | 1.582219766 | 1.80573E-10 |
| 292. | MPZL2 | 1.875289504 | 6.73374E-11 |
| 293. | LYNX1 | 1.776638303 | 6.82835E-08 |
| 294. | ALDH6A1 | 1.769734983 | 2.05395E-07 |
| 295. | GTF2H2C_2 | 1.558908948 | 1.0152E-08 |
| 296. | GTF2H2 | 1.558908948 | 1.0152E-08 |
| 297. | IDO2 | 1.699432821 | 2.80159E-05 |
| 298. | RNF14 | 1.535615116 | 1.53724E-08 |
| 299. | CAV1 | 1.51201243 | 6.16903E-13 |
| 300. | RAP1A | 10.07126733 | 1.6791E-20 |
| 301. | ALOX5 | 1.513235646 | 9.24483E-06 |
| 302. | QKI | 2.0104057 | 6.65981E-09 |
| 303. | CREBRF | 1.612724494 | 4.52294E-06 |

| | | | |
|---|---|---|---|
| 304. | BICD2 | 1.631791428 | 6.03455E-09 |
| 305. | SNORA63 | 1.635437546 | 6.53341E-09 |
| 306. | HSD11B1 | 1.507000609 | 2.21642E-05 |
| 307. | VAV3 | 1.535817367 | 3.38761E-06 |
| 308. | SETBP1 | 1.685737617 | 3.7977E-05 |
| 309. | HSPA2 | 1.546421213 | 3.31411E-08 |
| 310. | CHMP1B | 1.578302054 | 5.36051E-10 |
| 311. | SEMA3C | 1.563916334 | 2.34152E-07 |
| 312. | RPSA | 1.969168637 | 4.29798E-09 |
| 313. | KRT19 | 1.945656011 | 7.83438E-15 |
| 314. | SERP1 | 2.141631108 | 2.30384E-13 |
| 315. | DIXDC1 | 1.628119582 | 5.79281E-09 |
| 316. | CCNB1IP1 | 1.557353728 | 1.25541E-09 |
| 317. | FAM101B | 1.76599754 | 1.97531E-11 |
| 318. | SAMD11 | 1.575685928 | 2.39945E-10 |
| 319. | SLC35A2 | 1.519952951 | 6.97162E-08 |
| 320. | S1PR1 | 1.529545828 | 0.000148995 |
| 321. | TMEM50A | 1.566654081 | 2.14771E-11 |
| 322. | PCDH20 | 1.50897266 | 0.000752001 |
| 323. | AP1M2 | 2.106181842 | 1.01107E-10 |
| 324. | PAWR | 1.568577343 | 3.68146E-10 |
| 325. | FAM161A | 1.532534976 | 4.20973E-06 |
| 326. | ASPH | 1.639493293 | 3.16857E-08 |
| 327. | AHNAK2 | 2.3235811 | 5.44468E-12 |
| 328. | PTCH1 | 1.84339256 | 8.81189E-08 |
| 329. | HLA-DMA | 1.737845708 | 8.25525E-07 |
| 330. | PRR16 | 1.918647926 | 3.5073E-07 |
| 331. | CMSS1 | 1.524849298 | 1.2554E-09 |
| 332. | ID3 | 1.716016391 | 6.3844E-12 |
| 333. | RND3 | 1.502408017 | 1.89097E-09 |
| 334. | ADAMTS1 | 2.53994396 | 3.62238E-11 |
| 335. | HSPA5 | 1.553939138 | 2.74916E-06 |
| 336. | KIF21B | 1.800644443 | 2.73628E-08 |
| 337. | KIAA1143 | 1.769501928 | 3.62237E-09 |
| 338. | SLC4A8 | 1.532591632 | 0.000104975 |
| 339. | PANK3 | 1.691105501 | 4.90426E-09 |
| 340. | CDC37L1 | 1.509565476 | 6.59028E-06 |
| 341. | FBXL17 | 1.646596046 | 0.000187323 |
| 342. | LOC102725241 | 1.533512578 | 4.15317E-05 |
| 343. | GBP5 | 1.714074719 | 8.56097E-09 |
| 344. | ZNF138 | 1.561089588 | 9.53596E-05 |
| 345. | SERPINI1 | 1.519893251 | 9.61617E-06 |
| 346. | AJUBA | 1.510064319 | 2.9196E-07 |
| 347. | B4GALT4 | 1.64555776 | 9.67668E-11 |

| | | | |
|---|---|---|---|
| 348. | SEL1L3 | 1.659516914 | 6.97588E-10 |
| 349. | RBM47 | 1.67962597 | 2.0231E-09 |
| 350. | RNF167 | 1.536037389 | 2.22769E-07 |
| 351. | GDF15 | 1.623739279 | 1.04162E-09 |
| 352. | GINM1 | 2.005858727 | 3.43614E-13 |
| 353. | SLC37A2 | 1.550585773 | 1.42266E-06 |
| 354. | DENND1A | 1.5462783 | 1.875E-08 |
| 355. | GRB7 | 1.575369227 | 2.38571E-09 |
| 356. | CTDSP2 | 1.996057573 | 9.76308E-12 |
| 357. | IFRD1 | 2.240762897 | 1.3579E-13 |
| 358. | ZNF566 | 1.581602073 | 2.74107E-09 |
| 359. | YRDC | 1.65093883 | 5.21124E-11 |
| 360. | CHORDC1 | 1.528086982 | 4.94985E-05 |
| 361. | EIF5A2 | 1.911351788 | 3.76504E-09 |
| 362. | XXYLT1 | 1.872289249 | 4.99318E-09 |
| 363. | BCCIP | 1.807380171 | 5.59765E-09 |
| 364. | AK4 | 1.890878503 | 6.3823E-07 |
| 365. | AK2 | 1.628006734 | 5.05631E-07 |
| 366. | FAM25A | 1.795995 | 7.34995E-12 |
| 367. | SLC16A9 | 1.616914433 | 5.99404E-07 |
| 368. | UTP14C | 1.583082747 | 6.55043E-09 |
| 369. | CAPN1 | 1.682364135 | 7.31365E-08 |
| 370. | ZSCAN31 | 1.531066204 | 3.84952E-08 |
| 371. | NAA15 | 2.200777558 | 1.12259E-10 |
| 372. | ABL2 | 1.624317133 | 5.34936E-09 |
| 373. | KCTD12 | 2.516909484 | 2.63101E-10 |
| 374. | INHBE | 1.86178094 | 2.94616E-05 |
| 375. | ESR1 | 2.045036605 | 8.32899E-10 |
| 376. | SNHG12 | 1.812553192 | 7.24646E-06 |
| 377. | B4GALT5 | 1.515713064 | 7.45537E-11 |
| 378. | YME1L1 | 1.775698528 | 3.67253E-13 |
| 379. | ZNF681 | 1.563887426 | 2.33996E-07 |
| 380. | PHGDH | 1.819430151 | 1.83704E-12 |
| 381. | NEXN | 1.976813361 | 2.46783E-07 |
| 382. | TMEM182 | 1.521505976 | 3.51421E-05 |
| 383. | TKT | 2.281000348 | 2.38281E-09 |
| 384. | SMAD7 | 1.503050345 | 4.68812E-07 |
| 385. | AKR1C1 | 1.699837302 | 1.3509E-12 |
| 386. | CBS | 1.561908565 | 1.40561E-09 |
| 387. | HCCS | 1.590587788 | 1.90649E-07 |
| 388. | YWHAH | 1.678132542 | 3.64473E-11 |
| 389. | GTF2H2C | 1.558908948 | 1.0152E-08 |
| 390. | SEPN1 | 1.99565638 | 4.2392E-10 |
| 391. | IL31RA | 1.613939688 | 8.09321E-05 |

| | | | |
|---|---|---|---|
| 392. | DGCR2 | 3.513713755 | 1.42218E-13 |
| 393. | IRAK2 | 1.520817108 | 5.61745E-09 |
| 394. | AMTN | 1.531030829 | 5.17221E-08 |
| 395. | TSC22D3 | 1.903331251 | 3.49725E-12 |
| 396. | EBP | 1.506676826 | 4.09026E-08 |
| 397. | NAV3 | 1.646733012 | 0.000105184 |
| 398. | SNTB1 | 1.991257782 | 3.2001E-11 |
| 399. | ARHGAP29 | 1.68018101 | 2.17746E-08 |
| 400. | IER5L | 1.538864992 | 3.74668E-10 |
| 401. | SNORA62 | 1.969168637 | 4.29798E-09 |
| 402. | SEMA7A | 1.6823447 | 1.34355E-09 |
| 403. | CFL2 | 3.936654227 | 3.34471E-14 |
| 404. | PUM3 | 1.513672749 | 3.10134E-08 |
| 405. | TGM2 | 1.578568282 | 9.96566E-07 |
| 406. | AKNA | 1.562471636 | 2.36193E-05 |
| 407. | CLVS1 | 1.591749525 | 5.23976E-07 |
| 408. | ZNF781 | 1.851280281 | 1.55596E-09 |
| 409. | MRPL57 | 1.535686078 | 3.09554E-07 |
| 410. | SLC30A1 | 1.624305875 | 5.34233E-09 |
| 411. | NPL | 1.522775574 | 8.64402E-06 |
| 412. | MKL2 | 1.55887293 | 2.13E-08 |
| 413. | TMED7-TICAM2 | 1.68130718 | 2.01978E-08 |
| 414. | FAM25BP | 1.750163858 | 2.18372E-12 |
| 415. | DTX3L | 1.595855472 | 2.58963E-07 |
| 416. | PHF10 | 1.544143324 | 6.05942E-10 |
| 417. | GFRA1 | 1.538872103 | 8.49329E-06 |
| 418. | PDZK1P1 | 1.815394804 | 1.04051E-07 |
| 419. | FAM25C | 1.750163858 | 2.18372E-12 |
| 420. | FAM73A | 1.518261188 | 2.99388E-06 |
| 421. | CDK8 | 1.530995455 | 3.85291E-09 |
| 422. | NCAPD2 | 1.515909191 | 2.75807E-08 |
| 423. | NBPF10 | 1.741692578 | 7.96928E-08 |
| 424. | APPL1 | 1.540942825 | 7.48262E-08 |
| 425. | C11orf70 | 1.551872465 | 2.02782E-08 |
| 426. | RNF130 | 1.63537331 | 2.5559E-11 |
| 427. | PM20D2 | 1.502203224 | 1.04827E-06 |
| 428. | SDC1 | 1.884754566 | 2.76963E-14 |
| 429. | OXTR | 1.677047246 | 7.99641E-07 |
| 430. | CDKN2C | 1.576844067 | 2.8887E-08 |
| 431. | TACSTD2 | 1.652828077 | 5.64355E-11 |
| 432. | UCP2 | 1.860104061 | 8.40565E-10 |
| 433. | FAM20C | 1.815621319 | 3.02008E-11 |
| 434. | NAP1L1 | 2.054294948 | 1.54014E-13 |
| 435. | DDIT4 | 1.773672933 | 5.10552E-13 |

| | | | |
|---|---|---|---|
| 436. | RAD18 | 1.630619305 | 5.83857E-10 |
| 437. | RBM43 | 1.575846123 | 2.74758E-07 |
| 438. | GRB2 | 1.653423924 | 3.49087E-10 |
| 439. | CXCR4 | 1.831810164 | 5.89937E-05 |
| 440. | GMNN | 2.327078696 | 8.52291E-15 |
| 441. | GPCPD1 | 2.116626431 | 4.62287E-11 |
| 442. | UBE2L3 | 1.543151814 | 6.22433E-05 |
| 443. | HACD2 | 1.529012287 | 2.56384E-05 |
| 444. | PPIL1 | 1.544350266 | 1.05858E-08 |
| 445. | PON2 | 1.744064425 | 1.26046E-10 |
| 446. | LMO4 | 1.635021946 | 9.8936E-09 |
| 447. | NARF | 1.513938569 | 1.8313E-10 |
| 448. | PRKAR2B | 1.51077624 | 4.24506E-05 |
| 449. | C2orf88 | 1.939574242 | 5.06887E-10 |
| 450. | THBS1 | 2.681233132 | 9.40453E-16 |
| 451. | SRGN | 1.51439337 | 7.58142E-05 |
| 452. | EIF3CL | 3.392079541 | 2.28119E-08 |
| 453. | ZNF706 | 1.719004514 | 1.79748E-08 |
| 454. | LVRN | 1.534122124 | 5.80475E-06 |
| 455. | SLC16A3 | 1.734347912 | 0.001444191 |
| 456. | ALG5 | 1.502293469 | 1.39132E-09 |
| 457. | ASAH2B | 1.533512578 | 4.15317E-05 |
| 458. | SORL1 | 1.840834593 | 2.39232E-12 |
| 459. | PTP4A1 | 1.542371181 | 7.32383E-09 |
| 460. | DISP1 | 1.5588081 | 4.70028E-07 |
| 461. | MYO1C | 1.5116736 | 7.37789E-08 |
| 462. | POLR3K | 1.504902479 | 9.24488E-10 |
| 463. | IL1R1 | 1.936735135 | 2.90444E-10 |
| 464. | PTGER4 | 1.58994846 | 3.2812E-06 |
| 465. | TP53INP1 | 1.538250008 | 2.40638E-08 |
| 466. | SLC35F2 | 1.657838341 | 7.38237E-10 |
| 467. | PROM2 | 1.517934985 | 3.33125E-08 |
| 468. | BCAT2 | 1.890070437 | 1.31701E-11 |
| 469. | PNPT1 | 1.5822417 | 2.81044E-11 |
| 470. | TNFRSF12A | 1.653305501 | 5.65652E-13 |
| 471. | GALNT10 | 1.756459121 | 5.88309E-09 |
| 472. | SMCO4 | 1.830477449 | 3.34774E-11 |
| 473. | EIF5 | 1.588043025 | 3.81628E-11 |
| 474. | SH3BP4 | 1.614096313 | 1.6868E-10 |
| 475. | TAF5L | 1.826122229 | 4.14186E-09 |
| 476. | ZNF98 | 1.522803721 | 3.03158E-05 |
| 477. | GTF2H2B | 1.558908948 | 1.0152E-08 |
| 478. | PLSCR1 | 1.69581642 | 3.47109E-07 |
| 479. | HBP1 | 1.566426054 | 7.65356E-09 |

| | | | |
|---|---|---|---|
| 480. | FOS | 1.971759144 | 4.05126E-12 |
| 481. | HEATR3 | 1.539064115 | 1.8853E-07 |
| 482. | PERP | 1.559359244 | 3.67169E-06 |
| 483. | CTSC | 1.903546747 | 9.09635E-09 |
| 484. | IGFBP6 | 1.558105944 | 4.11355E-13 |
| 485. | RCC2 | 1.638035549 | 1.30386E-07 |
| 486. | SRM | 1.713714365 | 2.28781E-10 |
| 487. | ETS1 | 1.639364505 | 7.96642E-06 |
| 488. | ITGB8 | 2.074815904 | 3.61946E-12 |
| 489. | CTPS1 | 1.572278369 | 1.13921E-10 |
| 490. | FRMD6 | 1.579370885 | 5.71596E-10 |
| 491. | MPP1 | 2.404860707 | 1.94897E-12 |
| 492. | BRWD1 | 1.513032874 | 2.69622E-07 |
| 493. | CTDSP1 | 1.519538611 | 8.15937E-09 |
| 494. | GANAB | 1.614987875 | 3.38077E-07 |
| 495. | CDKAL1 | 1.712249968 | 1.8226E-07 |
| 496. | SEC14L2 | 1.612307215 | 8.3373E-07 |
| 497. | S100A14 | 1.507380185 | 0.00020963 |
| 498. | MED13 | 1.531855273 | 4.2144E-09 |
| 499. | KLHL2 | 2.157982751 | 3.84894E-11 |
| 500. | ARHGAP27 | 1.579469414 | 5.14499E-06 |
| 501. | CGNL1 | 1.697474615 | 1.06204E-08 |
| 502. | HMG20A | 1.805739774 | 5.30891E-11 |
| 503. | METTL21A | 1.663267254 | 5.27406E-09 |
| 504. | SERPINB5 | 1.660069144 | 4.54543E-10 |
| 505. | SPDL1 | 1.508596168 | 7.3526E-12 |
| 506. | VEZF1 | 1.543319399 | 1.67795E-10 |
| 507. | FAM167A | 2.676362126 | 3.26146E-15 |
| 508. | TGFBR1 | 1.596582017 | 4.33607E-11 |
| 509. | NUPR1 | 1.78469804 | 1.56653E-11 |
| 510. | XBP1 | 1.701208537 | 6.26755E-12 |
| 511. | RIOK3 | 1.721651732 | 4.6316E-12 |
| 512. | MTCH2 | 2.238791228 | 2.13002E-12 |
| 513. | CALCOCO1 | 1.681485883 | 5.83219E-06 |
| 514. | MAL | 1.838726199 | 2.62953E-14 |
| 515. | EFNA1 | 1.504356679 | 4.8012E-10 |
| 516. | GRB10 | 1.569534419 | 1.11441E-07 |
| 517. | NBPF1 | 1.741692578 | 7.96928E-08 |
| 518. | ZNF655 | 1.577299543 | 6.892E-06 |
| 519. | SERAC1 | 1.665863278 | 3.5975E-07 |
| 520. | LMCD1 | 1.543772325 | 9.97952E-07 |
| 521. | ASNS | 1.671577216 | 9.26548E-11 |
| 522. | HERPUD1 | 1.618813367 | 3.22986E-12 |
| 523. | WDR4 | 1.770201185 | 6.70583E-07 |

| | | | |
|---|---|---|---|
| 524. | GPATCH4 | 1.555440634 | 4.47168E-09 |
| 525. | RPS14 | 1.934525841 | 3.43518E-12 |
| 526. | OTULIN | 2.009964471 | 4.24192E-10 |
| 527. | RRS1 | 1.566784397 | 5.04962E-10 |
| 528. | TUFT1 | 2.490266253 | 1.00731E-10 |
| 529. | NRG1 | 2.014772112 | 2.1588E-10 |
| 530. | EMP1 | 1.552370942 | 3.43672E-07 |
| 531. | JADE2 | 1.50263714 | 1.77364E-07 |
| 532. | SMIM15 | 1.766083229 | 2.22516E-10 |
| 533. | DYX1C1 | 1.774427136 | 1.02534E-07 |
| 534. | FAM111B | 1.557839568 | 9.166E-05 |
| 535. | IL36G | 1.535363227 | 1.36317E-05 |
| 536. | TPD52 | 1.750479298 | 5.30612E-11 |
| 537. | USMG5 | 1.538680116 | 2.27474E-07 |
| 538. | B3GNT5 | 1.720462762 | 3.32936E-10 |
| 539. | MRPL24 | 1.556738548 | 6.23244E-11 |
| 540. | GJD3 | 1.546181842 | 1.05752E-07 |
| 541. | HIST1H2BK | 2.268496866 | 1.50394E-10 |
| 542. | NUP88 | 1.581514372 | 5.49755E-06 |
| 543. | KRT15 | 1.546867902 | 3.70848E-08 |
| 544. | CTH | 1.575977204 | 2.42647E-10 |
| 545. | IPO11 | 1.769264815 | 1.24738E-10 |
| 546. | ULBP1 | 1.663267254 | 5.88365E-06 |
| 547. | COQ10B | 1.593615222 | 1.44111E-06 |
| 548. | AKR1C2 | 1.676683054 | 4.81921E-12 |
| 549. | RAD23B | 1.944258438 | 0.010246897 |
| 550. | MOK | 1.55166093 | 1.1701E-07 |
| 551. | PRNP | 1.509565476 | 0.000174753 |
| 552. | ZNF385A | 1.760229247 | 3.81001E-07 |
| 553. | THBD | 2.296059455 | 1.73318E-13 |
| 554. | RIPK1 | 1.514851807 | 4.2384E-07 |
| 555. | SCNN1A | 1.759973045 | 2.35592E-09 |
| 556. | CCDC28A | 1.663132756 | 3.33714E-10 |
| 557. | CHST2 | 1.572739794 | 1.23845E-10 |

**Supplementary Table 2 High-scoring networks (score > 20) identified by Ingenuity Pathway Analysis® in SKOV3 cells. The top six of 25 networks are represented here**

| ID | Molecules in Network | Score | Focus Molecules | Top Diseases and Functions |
|---|---|---|---|---|
| 1 | BASP1,**BCCIP**,CHST2,CLIC3,**CRIM1**, CSRP1,**DNAAF4**,**EIF5A2**,**EPB41L5**,ESR1, HBP1,KIF3B,KLHL24,**LMO4**,MPP1,NCAPD2,**NUP88**,POLD4,**PPIF**,PPIL1,RFX2,SAMD11,SERPINB5,SLC1A4,**SLC44A2**,**SPDL1**,SRM,**TIPIN**,TKT,VEZF1,VPS36,WARS | 53 | 32 | Embryonic Development &Organ Development&Organ Morphology |
| 2 | ADK,**ANKRD1**,APPL1,**APPL2**,B4GALT5,BRWD1,CHMP1B,**DENND1A**,**DMKN**, EIF3CL,**EPHA4**,GDF15,GINM1,**GRB10**,INPP4B,**ITGB8**,**MYPN**,**PCDH20**,RAB31,**RBM24**,SEC14L2,SPG20,TACSTD2,TPD52,ULBP1,WASF2 | 38 | 26 | Cell Morphology&Cellular Assembly and Organization& Cellular Function and Maintenance |
| 3 | **AJUBA**,AMTN,ARHGAP27,ARPC5,**BICC1**, **CPA4**,DISP1,**DOCK2**,FAM20C,FOS,GMFB, LAPTM5,MPZL2,**MPZL3**,MYO5B,**NABP1**,**NBPF10** (includes others),**PLK2**,PROM2,QKI,RCC2,,SNX18,SOS2,STK17A,**XDH** | 36 | 25 | Cell Signaling&Organ Morphology& Organismal Development |
| 4 | ASNS,**CTGF**,DDIT4,**FRMD6**,HES1,ID2, ID3,ID4,MAP3K3,**NPL**,PEX5,PHGDH, PKDCC,RPAP3,RRM2,SLC33A1,SMAD7,SPARC,TBC1D8,TIMP3,TNS2,TP53INP1,**ZNF655** | 32 | 23 | Cellular Development&Cellular Growth and Proliferation, |
| 5 | **ARL6**,ARL6IP5,CAPN1,**CDC25A**,Cg,Ck2,**CTPS1**,DHRS3,DMRT3,FSH,**GGCT**,Gsk3,**HACD2**,HMG20A,KIF21B,Lh,MTMR9,Nfat (family),NMDA Receptor,**PAWR**,phosphatase,PIKFYVE,Pka,PNO1,**PTP4A1**,PTPase,**PTPRF**,PXYLP1, **RASSF2**,Sod,TMEM185A,tyrosine kinase,**UBASH3B**,UCP2,UTP14C | 32 | 23 | Carbohydrate Metabolism&Cell Morphology& Cellular Assembly and Organization |
| 6 | alcohol group acceptor phosphotransferase,**ARRDC4**,BTF3L4,Cbp/p300,**CDK8**,CK1,DBP,ENaC,FAM20B,FLOT1,**GTF2H2**,Holo RNA polymerase ,**JUN**,MED13,MED14,mediator, MEF2,MIR124,**NEXN**,NFAT(complex),Pp2b, **RAD23B**,SCNN1A,SEC24D,**SERP1**,**SGK1**, SLC30A1,SLC37A2,**SLC4A8**,TAF5L,TFIIH, | 30 | 22 | Molecular Transport& Cellular Function and Maintenance& Cell Signaling |

| |
|---|
| TRAP/Media,TSC22D3,UBIQUITIN LIGASE,**YRDC** |

The genes found to be differentially regulated and the number of such genes displayed in the “focus molecules” column that meet the cutoff and/or filter criteria were mapped to its corresponding gene object in IPA knowledge base (bold up-regulated, regular font down regulated).The score, generated using a p value calculation, indicates the likelihood that the assembly of a set of focus genes in a network could be explained by random chance alone. The database attributed general cellular functions to each network which was determined by interrogating the Ingenuity Pathway Knowledge base for relationships between the genes in the network and the cellular functions they impact.

**Table S3 The change of differential gene expression level can inhibit the activation of disease and function.**

| | Diseases or Functions Annotation | *P* | Z |
|---|---|---|---|
| 1 | Cell-To-Cell contact | 0.0000349 | 2.592 |
| 2 | Organismal Survival | 0.00000176 | 2.462 |
| 3 | Cellular Assembly and Organization | 0.000726 | 2.399 |
| 4 | Organismal Survival | 0.00000124 | 2.392 |
| 5 | Cell Death and Survival, Connective Tissue Development and Function | 0.000204 | 2 |
| 6 | Migration of microvascular endothelial cells | 0.000902 | -2.219 |
| 7 | Leukopoiesis | 0.00112 | -2.214 |
| 8 | Endocytosis | 0.000435 | -2.105 |
| 9 | Survival of organism | 0.000112 | -2.056 |

Z-score>2 represents a significant activation of the disease or function. Z-score<-2 represents a significant inhibition of the disease or function.
